# Human Prion Disease Surveillance in Washington State, 2006-2017

**DOI:** 10.1001/jamanetworkopen.2020.20690

**Published:** 2020-10-16

**Authors:** Liliana Sánchez-González, Ryan A. Maddox, Larissa C. Lewis, Janis E. Blevins, Elizabeth J. Harker, Brian S. Appleby, Marissa K. Person, Lawrence B. Schonberger, Ermias D. Belay, Chas DeBolt, Kathryn H. Lofy

**Affiliations:** 1 Washington State Department of Health, Shoreline; 2 Dengue Branch, Division of Vector Borne Diseases, Centers for Disease Control and Prevention, San Juan, Puerto Rico; 3 Division of High-Consequence Pathogens and Pathology, National Center for Emerging and Zoonotic Infectious Diseases, Centers for Disease Control and Prevention, Atlanta, Georgia; 4 National Prion Disease Pathology Surveillance Center, Case Western Reserve University, Cleveland, Ohio; 5 Hyland Software, Westlake, Ohio; 6 Texas Department of State Health Services, Austin

## Abstract

**Importance:**

Human prion disease surveillance is critical to detect possible cases of variant Creutzfeldt-Jakob disease and other acquired forms of prion disease in the United States. Results are presented here that describe 12 years of surveillance in Washington, the only US state that has reported the presence of classic bovine spongiform encephalopathy, an animal prion disease that has been shown to transmit to humans.

**Objective:**

To describe the current prion disease surveillance system in Washington and the epidemiological and clinical results of surveillance from 2006 through 2017.

**Design, Setting, and Participants:**

This cross-sectional study reports findings from the human prion disease surveillance system in place in Washington state from January 1, 2006, through December 31, 2017. Participants included Washington residents with a clinical suspicion of human prion disease or suggestive test results from the National Prion Disease Pathology Surveillance Center or with prion disease listed as a cause of death on the death certificate. Data for this report were analyzed from June 1, 2016, to July 1, 2020.

**Exposure:**

Human prion disease diagnosis.

**Main Outcomes and Measures:**

The main outcome was incidence of human prion disease cases, including identification of variant Creutzfeldt-Jakob disease.

**Results:**

A total of 143 human prion disease cases were detected during the study period, none of which met criteria for a variant Creutzfeldt-Jakob disease diagnosis. Among 137 definite or probable cases, 123 (89.8%) occurred in persons aged 55 years or older, with a median age at death of 66 years (range, 38-84 years). Most patients were White (124 [92.5%] among 134 with reported race), and slightly over half were male (70 [51.1%]). The average annual age-adjusted prion disease incidence was 1.5 per million population per year, slightly higher than the national rate of 1.2 per million. A total of 99 cases (69.2%) were confirmed by neuropathology. Sporadic prion disease was the most common diagnosis, in 134 cases (93.7%), followed by familial prion disease in 8 cases (5.6%). One iatrogenic prion disease case (0.7%) was also reported.

**Conclusions and Relevance:**

The findings of this cross-sectional study suggest that demographic characteristics of patients with prion disease in Washington are consistent with national findings. The slightly higher incidence rate may be due to the state’s enhanced surveillance activities, including close collaboration with key partners and educational efforts targeted toward health care providers. Results indicate that surveillance will continue to be beneficial for monitoring epidemiological trends, facilitating accurate diagnoses, and detecting variant Creutzfeldt-Jakob disease or other emerging human prion disease cases.

## Key Points

**Question:**

What are the results of human prion disease surveillance in Washington state?

**Findings:**

In this cross-sectional study using state surveillance data from 2006 to 2017, 143 human prion disease cases were detected, with an average annual age-adjusted incidence consistent with national reports. The majority of cases (94%) were sporadic, and no cases met criteria for a variant Creutzfeldt-Jakob disease diagnosis.

**Meaning:**

These findings indicate that prion disease surveillance in Washington state is beneficial for monitoring epidemiological trends, facilitating accurate diagnoses, and detecting variant Creutzfeldt-Jakob disease or other emerging human prion diseases should they occur.

## Teaser

This cross-sectional study describes the current prion disease surveillance system in Washington state and reports the epidemiological and clinical results of surveillance activities from 2006 through 2017.

## Introduction

Prion diseases, also called transmissible spongiform encephalopathies, are a group of rare, fatal, neurodegenerative diseases that occur in animals and humans. These diseases are characterized by the conversion of normal prion proteins into abnormal, pathogenic agents known as prions.^1,2,3^ This conversion process can be sporadic, related to a genetic mutation, or induced by the uptake of the pathogenic prions.^3,4^ The accumulation of prions is associated with neuronal injury leading to spongiform changes in the central nervous system.^5^

The most common human prion disease (HPD) is Creutzfeldt-Jakob disease (CJD), with an age-adjusted incidence of 1.2 cases per million population per year in the United States, similar to the incidence reported in other countries.^6,7^ Sporadic CJD accounts for approximately 85% of CJD cases and familial CJD for 10% to 15%.^3^ Other less common prion diseases include fatal familial insomnia, sporadic fatal insomnia, Gerstmann-Straüssler-Scheinker disease, variably protease-sensitive prionopathy, and acquired prion disease.

In 1996, a new, acquired HPD, termed variant CJD (vCJD), was described in the United Kingdom.^8^ It was linked to the consumption of prion-contaminated beef or beef products from cattle afflicted with bovine spongiform encephalopathy (BSE).^9^ Later, several patients with vCJD were determined to have contracted their illness through receipt of blood from donors who subsequently developed vCJD.^10,11^ As of January 2020, 232 cases of vCJD have been described around the world.^12^ This number includes 4 patients who were diagnosed in the United States but were likely exposed to the infectious agent outside the country.^13^ Variant and iatrogenic (ie, health care–acquired) CJD accounts for less than 1% of the total number of HPD cases.

In December 2003, a dairy cow imported from Canada into the state of Washington was diagnosed with BSE. Beef from the slaughtered cow had been processed for human consumption, and a recall of all beef from cattle slaughtered the same day at the involved slaughter plant was requested.^14^ In response to this incident, the Washington State Department of Health (WA DOH) established an enhanced HPD surveillance system the following year and has maintained a centralized database of all suspected cases in the state since that time. Human prion disease has been a distinct notifiable condition in Washington (Washington Administrative Code 246-101-101) since February 2011. Once clinically diagnosed, cases are to be reported within 3 business days to public health authorities (ie, local health jurisdictions).^15^ Before 2011, HPD was notifiable under the category of “other rare diseases of public health significance.”

The objectives of surveillance activities in Washington are (1) to establish background incidence rates and monitor trends in the epidemiology of HPD in the state, (2) to detect the possible emergence of vCJD or a possible new HPD, (3) to detect and help prevent potential iatrogenic CJD, and (4) to facilitate accurate HPD diagnoses. To aid in accomplishing these objectives, and in collaboration with the US Centers for Disease Control and Prevention (CDC), WA DOH personnel work closely with the National Prion Disease Pathology Surveillance Center (NPDPSC) at Case Western Reserve University, which is supported by the CDC and sponsored by the American Association of Neuropathologists. A major purpose of the NPDPSC is to provide state-of-the-art diagnostic testing for clinically suspected HPD cases.^16^

This article describes the current HPD surveillance system in place in Washington and the epidemiological and clinical results of surveillance activities during the years 2006 through 2017.

## Methods

This cross-sectional study was conducted using data obtained through the HPD surveillance system in Washington state for HPD cases with date of death from January 1, 2006, through December 31, 2017. Prion disease surveillance is a component of regular WA DOH duties; associated activities are not considered research and are not reviewed by an ethics committee. The presentation and discussion of study findings followed the Strengthening the Reporting of Observational Studies in Epidemiology (STROBE) reporting guideline.

### Case Finding

The WA DOH receives prion disease reports from 3 main sources: the NPDPSC, death certificate data, and health care professionals, including physicians and infection control professionals. On rare occasions, notifications from the general public have also been received.

The NPDPSC provides a WA DOH prion epidemiologist (including L.S.-G. or L.L.) with a copy of the results of brain autopsies and tests performed at the center for Washington residents or those ordered by Washington health care professionals. Among the possible tests are cerebrospinal fluid (CSF) Tau protein, CSF 14-3-3 protein, second-generation real-time quaking-induced conversion (RT-QuIC), brain biopsy, and blood *PRNP* genotyping. For some cases, the center may arrange for expert reviews of available magnetic resonance imaging results. The prion epidemiologist combines these NPDPSC data with available WA DOH information from case reports and investigations.

All patients in Washington with at least 1 positive test result (ie, positive 14-3-3, positive RT-QuIC, or tau protein level >1150 mg/mL) are investigated. Patients for whom clinical or epidemiological suspicion persists despite negative results are discussed with the CDC to determine the need for additional follow-up. The WA DOH conducts most of the investigations, although some of the 35 local health jurisdictions in the state conduct their own.

The WA DOH prion epidemiologist maintains routine and frequent communication with the State Vital Statistics epidemiologist. All death certificates with literal text and/or codes corresponding with HPDs (eg, Creutzfeldt-Jakob, fatal familial insomnia, prion disease, Gerstmann-Sträussler-Scheinker) are reviewed to determine whether the case is in the prion epidemiologist’s database and to establish whether further follow-up is necessary.

### Case Investigation

Data on demographics, clinical presentation and course, and infection prevention and control are collected. Race and ethnicity are obtained from the demographics section of available medical records, if noted, with options being defined by the hospitals and medical institutions; otherwise, these data are obtained from the death certificate. These characteristics were assessed because previous studies have shown differences in prion disease incidence by race.^6,17^

After notification of a suspected case, the prion epidemiologist obtains and reviews medical records and tests results. Facilities where the patient had been hospitalized are contacted, and interviews with clinicians, infection control professionals, and/or families are pursued to collect information on clinical presentation and risk factors for acquired prion disease, such as previous neurosurgery or receipt of a cadaver-derived pituitary hormone. Information regarding prion diseases and infection control measures are shared with infection control professionals.

The prion epidemiologist encourages health care professionals to discuss the possibility of autopsy with the patient’s caregivers when appropriate to obtain neuropathological confirmation of the diagnosis. Information regarding the NPDPSC’s autopsy service is given to the health care professional; information regarding other services available at the NPDPSC (eg, genetic testing for familial prion disease) and support services (eg, CJD Foundation) is given to families.

Cases of CJD are classified as sporadic (definite, probable, or possible), familial, or iatrogenic according to the current CDC Diagnostic Criteria.^18^ Cases lacking histopathological confirmation, genetic testing, and information indicating an iatrogenic source of infection or familial disease are classified as sporadic by default. Cases with insufficient information available for designation as definite, probable, or possible CJD are classified as physician-diagnosed CJD, provided CJD is listed as a cause of death on the death certificate and a prion disease diagnosis cannot be ruled out. Cases are followed until a prion disease diagnosis is excluded or confirmed or the patient dies. If biopsy or autopsy is performed, the follow-up continues until final results of brain tissue tests are received, a type of prion disease is established, and the case is closed.

Cases in which the patient is younger than 55 years or those with unusual clinical presentation or diagnostic results, concerning exposures, or any other special characteristics are discussed with the CDC and the NPDPSC in order to determine the need for further testing or public health measures.

### Data Collection and Statistical Analysis

All investigated cases (confirmed or not) are included in a confidential line list maintained by the WA DOH prion epidemiologist. Cases are also included in the Public Health Information Management System database. This database contains demographic, clinical, and infection control information regarding patients with notifiable conditions reported to WA DOH. When available, medical records for the HPD cases reported and investigated in Washington with date of death between January 1, 2006, and December 31, 2017, were reviewed and the abstracted information analyzed. Data analysis was conducted from June 1, 2016, to July 1, 2020.

Tau protein results were available from 2007 through 2017, second-generation RT-QuIC results were available starting in April 2015, and 14-3-3 results were available for the full 12 years included in this study. Incidence rates and demographic statistics were calculated based on cases classified as sporadic prion disease (definite or probable), familial prion disease, or iatrogenic CJD. Information regarding onset, clinical presentation, CSF test results, electroencephalography results, brain imaging studies, and pathology results were analyzed using SAS statistical software, version 9.4 (SAS Institute, Inc). Average annual age-adjusted incidence rates were calculated using the year 2000 as the standard population. Statistical significance was set at *P* < .05, and a 2-sided *P* value was calculated from the 95% CIs of the rate difference assuming a normal distribution.

## Results

A total of 143 HPD cases in Washington with date of death during 2006 through 2017 were detected (Table 1) and classified according to CDC criteria. Most of the cases (n = 134 [93.7%]) were classified as sporadic prion disease, 8 cases (5.6%) as familial prion disease, and 1 case (0.7%) as iatrogenic CJD. No cases of vCJD were identified. The average annual age-adjusted prion disease incidence was 1.5 per million population per year. For the periods 2006-2009, 2010-2012, 2013-2015, and 2015-2017, the incidence was 1.6, 1.3, 1.5, and 1.5 cases per million population, respectively, demonstrating stability. Demographic characteristics are described in Table 2. Among 137 definite or probable cases, 123 (89.8%) occurred in persons aged 55 years or older, with a median age at death of 66 years (range, 38 to 84 years). Most patients were White (n = 124 [92.5%] among 134 with reported race), and slightly over half were male (n = 70 [51.1%]). Average annual age-adjusted incidence between men and women (1.6 vs 1.4 per million, respectively) was not different (*P* = .35). The highest average annual age-specific incidence rate, 9.1 per million, was observed in those aged 75 to 84 years.

**Table 1.** Human Prion Disease Cases Reported in Washington State, 2006-2017

Disease category	Definite	Probable	Possible	Physician-diagnosed	Total
Sporadic	92	36	4	2	134
Familial	8	NA	NA	NA	8
Iatrogenic	1	NA	NA	NA	1
Total	101	36	4	2	143

Abbreviation: NA, not applicable.

**Table 2.** Demographic Characteristics of 137 Decedents With Definite and Probable Human Prion Disease in Washington State, 2006-2017^a^

Characteristic	No. (%)
Total	137 (100.0)
Age at death, y	
<55	14 (10.2)
55-64	46 (33.6)
65-74	48 (35.0)
75-84	29 (21.2)
Sex	
Male	70 (51.1)
Female	67 (48.9)
Race/ethnicity^b^	
White	124 (92.5)
Asian	5 (3.7)
Black	4 (3.0)
Native American	1 (0.7)
Initial reporter	
National Prion Disease Pathology Surveillance Center	104 (75.9)
Physician or health care facility	21 (15.3)
Death certificate	6 (4.4)
Funeral home	1 (0.7)
Other	5 (3.6)

^a^ Four possible cases and 2 physician-diagnosed cases excluded.

^b^ Race/ethnicity unknown in 3 cases.

Of the total 134 sporadic prion disease cases, 92 (68.7%) were neuropathologically confirmed (ie, definite cases); these cases included sporadic CJD (n = 90 [97.8%]), sporadic fatal insomnia (n = 1 [1.1%]), and variably protease-sensitive prionopathy (n = 1 [1.1%]) (Figure). Among the 90 definite sporadic CJD cases, 38 (42.2%) were the MM1 subtype, 10 (11.1%) were MV1, 10 (11.1%) were VV2, 8 (8.9%) were MM1-2, 8 (8.9%) were MV1-2, 7 (7.8%) were MV2, 5 (5.6%) were VV1-2, and 3 (3.3%) were MM2; 1 sporadic CJD case for whom frozen autopsy tissue was unavailable for analysis was considered by NPDPSC to be consistent with the MM(MV)1 subtype. Of the remaining sporadic prion disease cases (n = 42), most were classified as probable CJD (n = 36 [85.7%]). Test results for second-generation RT-QuIC in CSF were available for 10 of the probable CJD cases, 9 of which were positive, indicating likely true prion disease given the test’s very high specificity.^19^ Four of the 42 cases (9.5%) met criteria for possible CJD, and the remaining 2 cases lacked sufficient information for classification and were designated as physician-diagnosed cases; both of these patients were older than 60 years, making a vCJD diagnosis unlikely.

**Figure.**
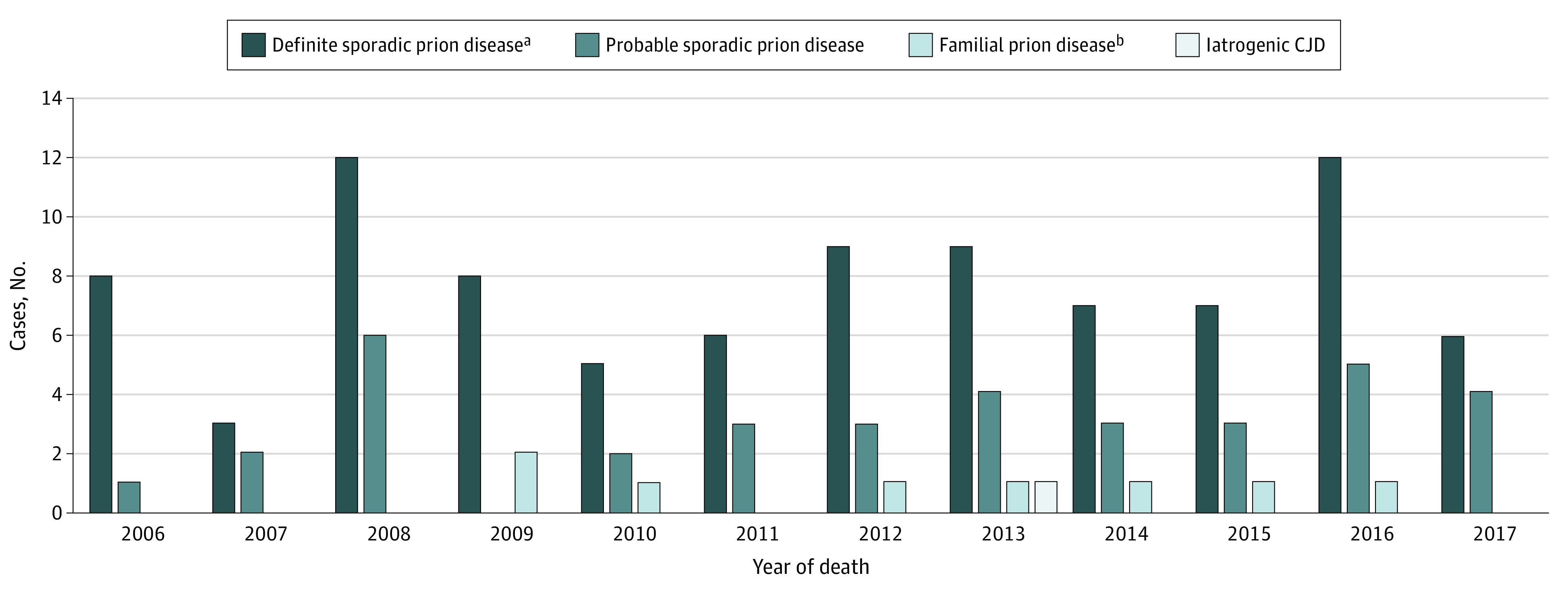
Definite and Probable Human Prion Disease Cases by Type and Year of Death, Washington State, 2006 to 2017 Results include 137 individuals. CJD indicates Creutzfeldt-Jakob disease. ^a^Sporadic prion disease includes sporadic CJD, variably protease-sensitive prionopathy, and sporadic fatal insomnia. ^b^Familial prion disease includes familial CJD and Gerstmann-Sträussler-Scheinker disease.

Of the 8 familial prion disease cases, 7 were familial CJD, and 1 was Gerstmann-Straüssler-Scheinker disease. All familial cases were classified as definite, including 2 that lacked neuropathologic testing but met clinical criteria and had a genetic mutation and/or positive family history. Three of the familial CJD cases had the E200K mutation; the remaining 4 cases had T183A, T188R, or D178N mutations, or a 5 octapeptide insert. The Gerstmann-Straüssler-Scheinker case had a 9 octapeptide insert.

The iatrogenic CJD case was associated with cadaver-derived human growth hormone administration during childhood and is part of an ongoing US outbreak of human growth hormone–associated CJD.^20^ It also had the unusual, distinct pathological phenotype reported as likely specific for iatrogenic CJD.^20,21,22^

Cases were reported from 26 of 35 local health jurisdictions (74.3%). There was no clear geographic clustering. One-third of the cases (n = 55 [38.4%]) occurred among residents of King and Pierce counties, which include the cities of Seattle and Tacoma and represent 41% of the state’s population.^23^

A majority of all prion disease cases (n = 99 [69.2%]), including 9 of the 15 cases (60.0%) in patients younger than 55 years at death, were neuropathologically confirmed by brain biopsy (n = 3), autopsy (n = 89), or both (n = 7). The annual percentage of cases confirmed by neuropathology ranged from 56.3% to 90.0%. Of the 99 confirmed cases, 85 (85.9%) had a CSF 14-3-3 protein result available; of those, 67 (78.8%) had a positive test result, whereas 10 (11.8%) were ambiguous and 8 (9.4%) were negative. Seven of the 8 confirmed cases with a negative 14-3-3 protein result also had the Tau protein test performed; 2 had a Tau protein level greater than 1150 mg/mL, and 5 did not.

Case-defining symptoms and signs reported for sporadic prion disease patients are summarized in Table 3. All patients presented with rapidly progressive dementia. Cerebellar abnormalities were reported in 103 cases (75.2%), and approximately half of patients reported extrapyramidal abnormalities (n = 68 [49.6%]) and visual abnormalities (n = 61 [44.5%]). Akinetic mutism and pyramidal signs were the least commonly reported clinical features. The WA DOH surveillance activities described in the Methods continue uninterrupted, and no vCJD diagnoses have been made in Washington after the time frame of this study.

**Table 3.** Case-Defining Clinical Features for Definite and Probable Human Prion Disease Cases at Any Time of Disease, Washington State, 2006-2017^a^

Clinical finding	No. (%)		
	Definite (n = 101)	Probable (n = 36)	Total (n = 137)
Rapidly progressive dementia	101 (100.0)	36 (100.0)	137 (100)
Cerebellar abnormalities	72 (71.3)	31 (86.1)	103 (75.2)
Extrapyramidal	41 (40.6)	27 (75.0)	68 (49.6)
Visual abnormalities	51 (50.5)	10 (27.8)	61 (44.5)
Myoclonus	35 (34.7)	21 (58.3)	56 (40.9)
Pyramidal	14 (13.9)	9 (25.0)	23 (16.8)
Akinetic mutism	11 (10.9)	12 (33.3)	23 (16.8)

^a^ Four possible cases and 2 physician-diagnosed cases excluded.

## Discussion

Since the identification of a BSE-positive dairy cow in the state in 2003, WA DOH, in collaboration with the CDC and the NPDPSC, has been conducting HPD surveillance. During the 12-year period evaluated in this cross-sectional study, the vast majority of HPD cases identified were classified as sporadic CJD, and no vCJD cases were detected. The average annual age-adjusted incidence, 1.5 cases per million population, was stable during this period with no apparent geographical clustering. Higher incidence rates were reported among decedents who were male, White, and aged 55 years or older, congruous with national findings.^6^ The finding of an average annual age-adjusted incidence slightly higher than the national rate, 1.2 per million, may be at least partially explained by the enhanced prion disease surveillance conducted in Washington, which has led to improved case detection. Efforts have been made in the state to increase awareness among neurologists and other medical providers regarding prion disease and the services available at the NPDPSC for diagnosis and confirmation purposes.

The median age at death (66 years) is comparable to national findings and findings from other industrialized countries.^24,25,26^ As expected, the vast majority of cases occurred among persons aged 55 years or older. The proportion of people aged 65 years or older in Washington increased from 12.3% in 2010 to 16.2% in 2019^23^; as the state population continues to age, a corresponding increase in the number of HPD cases is expected. According to official estimates for 2010,^24^ 82.3% of Washington state residents are White, 7.3% are Asian, 3.7% are Black, and 1.8% are American Indian/Alaska Native. Consistent with previous studies,^2,6^ White decedents were overrepresented in our findings, comprising 92.5% of the definite and probable HPD cases.

Since 2003, 5 additional cases of BSE, all atypical, have been identified in other states; no further BSE cases have been reported in Washington, which remains the only state where classic BSE has been found.^14^ This fact, combined with the recall of beef products in Washington after the BSE-infected cow’s slaughter, increases the importance of the state’s human prion disease surveillance.

Recently, concerns about the potential for chronic wasting disease (CWD), a prion disease of deer, elk, and moose, to transmit to humans have also been raised.^27^ Chronic wasting disease has not yet been found in Washington despite extensive surveillance conducted between 2001 and 2012, when more than 5000 animals were tested without finding any positive results. Currently, the Washington Department of Fish and Wildlife conducts surveillance targeting only animals with clinical signs of CWD. Should CWD be found in Washington in the future, ongoing HPD surveillance will be beneficial in assessing whether there is any connection between animal and human disease.

### Limitations

A limitation of this study is that only partial medical records were available for some patients, and the complete clinical presentation of the disease, including initial signs and symptoms, was sometimes difficult to ascertain. This limitation could potentially influence case classification, because some actual criteria and findings may not have been considered. In addition, the highly specific second-generation RT-QuIC CSF test was not regularly used by NPDPSC until 2015. Most cases, however, had neuropathologic confirmation. Collaboration with the NPDPSC, which performs the majority of premortem tests, enables a timely notification to WA DOH of suspected cases in the state and thus contributes to the number of subsequent autopsies performed.

## Conclusions

In this cross-sectional study of Washington state’s HPD surveillance system, findings suggest that the demographic characteristics of patients with prion disease between 2006 and 2017 were consistent with national findings. Despite a statewide prion disease surveillance program being in place since 2004, neither vCJD nor another new prion disease has been detected in the state. Given the long incubation periods associated with prion diseases, ongoing vigilance and collaboration with surveillance partners continue to be necessary.
